# Hepatic adenylate cyclase 3 is upregulated by Liraglutide and subsequently plays a protective role in insulin resistance and obesity

**DOI:** 10.1038/nutd.2015.37

**Published:** 2016-01-25

**Authors:** Y Liang, Z Li, S Liang, Y Li, L Yang, M Lu, H F Gu, N Xia

**Affiliations:** 1 Department of Endocrinology and Metabolism, First Affiliated Hospital of Guangxi Medical University, Nanning, China; 2 Department of Oncology-Pathology, Karolinska University Hospital, Karolinska Institute, Stockholm, Sweden; 3 Rolf Luft Research Center for Diabetes and Endocrinology, Department of Molecular Medicine and Surgery, Karolinska University Hospital, Karolinska Institute, Stockholm, Sweden

## Abstract

**Objective:**

Recent studies have demonstrated that adenylate cyclase 3 (AC3) has a protective role in obesity. This gene resides at the pathway with glucagon-like peptide (GLP)-1. Liraglutide is a GLP-1 analog and has independent glucose and body weight (BW)-reducing effects. In the present study, we aimed to examine whether hepatic AC3 activity was regulated by Liraglutide and to further understand the effect of AC3 in reduction of BW and insulin resistance.

**Subjects:**

The diabesity and obese mice were induced from db/db and C57BL/6 J mice, respectively, by high-fat diet. Liraglutide (0.1 mg kg^−1^ per 12 h) was given to the mice twice daily for 12 weeks. C57BL/6 J mice fed with chow diet and obese or diabesity mice treated with saline were used as the controls. Hepatic AC3 gene expression at mRNA and protein levels was analyzed with real-time reverse transcription-PCR and western blot. Fasting blood glucose and serum insulin levels were measured and followed insulin resistance index (HOMA-IR) was evaluated according to the homeostasis model assessment.

**Results:**

After administration of Liraglutide, BW and HOMA-IR in obese and diabesity mice were decreased, whereas hepatic AC3 mRNA and protein expression levels were upregulated. The AC3 gene expression was negatively correlated with BW, HOMA-IR and the area ratio of hepatic fat deposition in the liver.

**Conclusions:**

The present study thus provides the evidence that hepatic AC3 gene expression is upregulated by Liraglutide. The reduction of BW and improvement of insulin resistance with Liraglutide may be partially explained by AC3 activation.

## Introduction

Obesity and type 2 diabetes (T2D) are public health problems that have reached epidemic proportions in the world.^1^ The increase in the prevalence of T2D parallels that of obesity. This dual epidemic has been called as ‘diabesity'.^2^ Therefore, it is of importance to develop the pharmacological agents suitable for treatment of both T2D and obesity. Since 2010, Liraglutide, a glucagon-like peptide-1 (GLP-1) receptor analog, has been used as an injectable drug prescribed for the treatment of T2D,^3^ because this drug has a prolonged half-life and can be administered once daily to improve the control of blood glucose levels in adults with T2D.^4, 5, 6^ Interestingly, clinical observation has demonstrated that Liraglutide helps body weight (BW) reduction in T2D patients.^7^ This additional benefit of Liraglutide related to the regulation of BW is unexpected in the original objective for developing this drug and the underlying mechanism is still not fully known.

Adenylyl cyclases (ACs) are enzymes, which catalyze the synthesis of 3′–5′ cyclic adenosine monophosphate from ATP. Cyclic adenosine monophosphate is an important second messenger and mediates downstream activity of protein kinase A and subsequently regulates insulin secretion in β-cells of pancreatic islets. In mammals, there are nine closely related isoforms of ACs, and AC3 is the third member and calcium-dependent isoform.^8, 9, 10^ Previous studies shown that the AC3 gene expression at mRNA levels was upregulated in pancreatic islets of Goto-Kakizaki (GK) rat.^11^ GK rat is a hereditary non-obese animal model of T2D and exhibits a markedly reduced glucose-induced insulin release.^12^ The AC3 activity in the liver of ob/ob mice was increased compared with lean control mice.^13^ Data from these animal experiments suggested that AC3 may have a role in the pathogenesis of T2D and obesity. In 2007, Nordmen *et al.* conducted a genetic association study in Swedish T2D patients and obese subjects and reported that the AC3 genetic polymorphisms were associated with body mass index with the protective effects.^14^ This finding was replicated by a genetic association study in a Chinese population and confirmed by a genome-wide association study in European Caucasians.^15, 16^ Furthermore, Wang *et al.* developed AC3-deficient mice and found that AC3 knockout mice become obese when aging mainly due to increased fat mass and larger adipocytes.^17^ Obviously, AC3 has a role in the regulation of BW.^18^

As AC3 and GLP-1 reside in the same signal transduction pathway, where cyclic adenosine monophosphate is catalyzed by AC3 and generated by activation of GLP-1,^19, 20^ we thus have a hypothesis that the AC3 activity may be increased with the administration of Liraglutide and subsequently results in the reduction of BW. To test our hypothesis, in the present study, we first employed obese and diabesity mice, and then analyzed hepatic AC3 gene expression at both mRNA and protein levels before and after Liraglutide treatment. Data from this study may provide evidence for better understanding the effects of Liraglutide in reduction of BW and improvement of insulin resistance via AC3 activation.

## Materials and methods

### Establishment of diabesity and obese mice

In this study, all experimental animals were purchased from Cavensla Laboratory Animal Technology Co. (Changzhou, China) and were maintained at the Animal Experiment Center of Guangxi Medical University, China. Experiments were begun using 4-week-old C57BL/6 J and db/db mice. The mice were housed in individual cages with a 12-h light/dark cycle, where they had free access to standard chow and water. After 1 week, 24 C57BL/6 J mice (12 males and females each) were randomly divided into three groups. In the control group (Cont, *n*=8, 4 males and females each), the mice were fed with a normal diet. The rest mice (*n*=16, 8 males and females each) were given with high-fat diet (HFD; 34.9% fat and 26.2% protein) for 12 weeks to generate the obese mice. In addition, 16 db/db mice (8 males and females each) were also given with HFD for 12 weeks to generate the diabesity mice. BWs and blood glucose levels were measured weekly. Mice with fasting blood glucose (FBG) levels >13.9 mmol l^−1^ (250 mg dl^−1^) for three consecutive days were considered to be diabetic.^21^ Mice with BW exceeding 20% of the standard weight were considered as obese. After successfully establishing the models, all mice were fed a normal diet.

The obese and diabesity mice were randomly divided into different groups as follows: obese mice with saline (O+S), obese mice with Liraglutide treatment (O+L), diabesity mice with saline (OD+S); diabesity mice with Liraglutide treatment (OD+L). The O+L and OD+L groups were treated with subcutaneous injections of the GLP-1 analog Liraglutide at a dose of 0.1 mg kg^−1^ per 12 h. The O+S and OD+s groups were treated with subcutaneous injections of the same dosage of saline. At 12 weeks, mice were fasted overnight and anesthetized with sodium pentobarbital (50 mg kg^−1^ i.p.). The blood samples were obtained from the angular vein. Plasma was separated by centrifugation at 4 °C and was stored at −20 °C until assayed. The liver tissue samples were immediately dissected, frozen in liquid nitrogen and stored at −80 °C until further analysis.

All animal experiments and care procedures were conducted in conformity with the Guidelines of the Animal Care and Use Committee of Guangxi Medical University, Nanning, China.

### Measurements of serum insulin and blood glucose levels

Serum insulin levels were measured with a mouse insulin ELISA kit (North Biotechnology Research Institute, Beijing, China). Blood glucose levels were detected using a glucose meter (Johnson&Johnson, New Brunswick, NJ, USA). Homeostasis model of assessment was used to assess insulin resistance (HOMA-IR). The HOMA-IR was calculated as [fasting insulin (mU l^−1^) x fasting glucose (mmol l^−1^)] /22.5.^22^

### Real-time reverse transcription-PCR

Gene ID number of the *adcy*3 gene in mouse is 104111and the *adcy*3 mRNA sequence is NM_138305.3. Total RNAs were isolated from the liver tissues, and cDNAs were synthesized using a Reverse Transcriptase Kit (Thermo Scientific, Waltham, MA, USA). The primer sequences of AC3 sense and antisense are 5′-GGACACGCTCACAAACATC-3′ and 5′-GCCACATTGACCGTATTGC-3′. As an internal control, the glyceraldehyde 3-phosphate dehydrogenase (gapdh) mRNA levels were analyzed using the following primers: sense 5′-ATCACTGCCACCCAGAAG-3′ and antisense 5′-TCCACGACGGACACATTG-3′. PCR experiments were conducted in a light cycler ABI 7500 (Applied Biosystems, Foster City, CA, USA) at 95 °C for 10 min, followed by 40 cycles of 95 °C for 15 s and 60 °C for 45 s. The relative copy numbers were calculated using the threshold crossing point (Ct) in the Light Cycler software, combined with the ΔΔCt calculations.

### Western blotting

The amino-acid sequence of adcy3 protein in mouse is NP_612178.2. Western blot analysis was performed as previously described.^23^ Isolated liver tissues were homogenized with the homogenization buffer on ice. Protein concentrations were measured with a bicinchoninic acid assay kit (Thermo Scientific) according to the manufacturer's instructions. Samples (40–60 mg) were boiled at 95 °C for more than 5 min and were then placed on ice and loaded onto 7.5% polyacrylamide gels. The gels were transferred to a nitrocellulose membrane (Millipore, Billerica, MA, USA) and were blocked with 5% milk in phosphate-buffered saline with 0.05% Tween 20 for 1–2 h. Blots were incubated with polyclonal AC3 antibody (1:1000, Santa Cruz Biotechnology, Inc., Dallas, TX, USA) overnight at 4 °C, followed by horseradish peroxidase-conjugated goat anti-rabbit IgG for 1 h at room temperature. Blots were developed with an enhanced chemiluminescence detection reagent kit (Millipore).

### Histological and morphometric analyses

The hepatic histologic changes were observed with hematoxylin and eosin (H&E) and Masson and Oil Red O staining. For H&E or Masson staining, liver tissues were dehydrated through serial alcohol and were cleared in xylene. The specimens were embedded in paraffin, cut in 5 μm sections and stained with H&E or Masson trichrome. For Oil Red O staining, liver tissues were sliced and snap-frozen in isopentane-cooled liquid nitrogen before cutting into 10 μm sections with a cryostat. Sections were fixed with 4% paraformaldehyde and placed in absolute propylene glycol for 5 min, then stained in pre-warmed Oil Red O solution for 15 min at 60 °C followed by differentiation with 85% propylene glycol and brief counterstaining. A digital Olympus BX-51 microscope (× 400; Olympus Corporation) was used to image the sections. Quantification of the area ratio of hepatic fat deposition to liver tissue measured by Oil Red O staining in each group was performed using Image Pro Plus 6.0 (Media Cybernetics Media Cybernetics L.P. 8484, Silver Spring, MD, USA) software.^24^ To evaluate the degree of lipid accumulation (steatosis score), we categorized the tissue into four grades, as follows:^25^ no lipid droplets (score=0); lipid droplets in <33% of hepatocytes (score=1); lipid droplets in 33–66% of hepatocytes (score=2) and lipid droplets in >66% of hepatocytes (score=3). The classification of fibrosis in nonalcoholic fatty liver disease by the Nonalcoholic Steatohepatitis Clinical Research Network (NASH CRN)^26^ is shown in Table 1. Two independent observers who were blinded to the physical outcome or other biological and pathological data for each sample evaluated all histological slides.

### Statistical analysis

Statistical power in the present study was ~90% according to the size of animals. Quantitative data are shown as the means ± s.d. Significant differences were analyzed using Student's *t*-test, one-way analysis of variance (ANOVA) or Pearson's correlation, where appropriate. When Student's *t*-test or one-way ANOVA analysis methodology was employed, the homogeneity of variance test was used. The value of *P*<0.05 was considered to be statistically significant. All analyses were performed using SPSS 17.0 (SPSS Inc., Chicago, MI, USA).

## Results

### BW, FBG and serum insulin levels in mice before and after Liraglutide treatment

**Figure 1:**
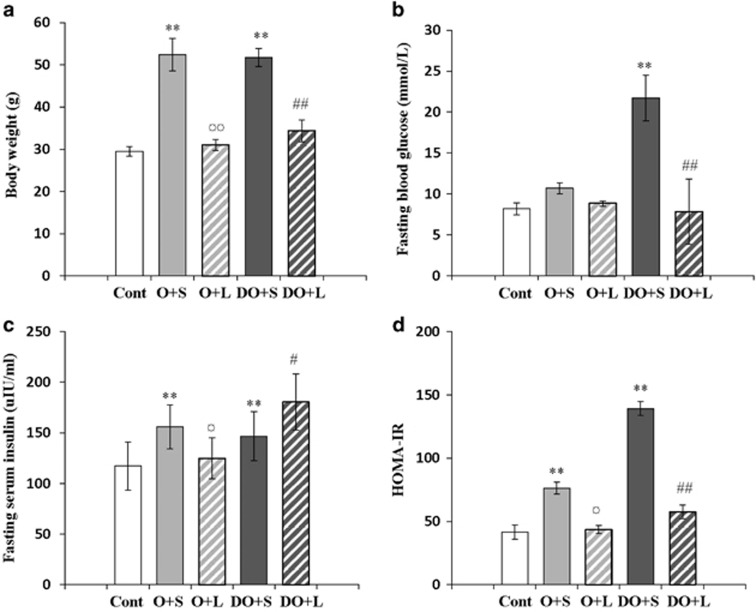
Body weight, fasting blood glucose and serum insulin levels in mice before and after Liraglutide treatment. The changes of body weight (g), fasting blood glucose and serum insulin levels in mice before and after Liraglutide treatment are summarized in **a**–**c**, respectively. HOMA-IR index is represented in **d**. **P<0.001 versus the control group before Liraglutide treatment; ^¤¤^*P*<0.001 test between O+S and O+L; ^##^*P*<0.001 between DO+S and DO+L; ^¤^*P*<0.05 and ^#^*P*<0.05. Abbreviations: Cont, the control group of non-diabetic and lean mice; O+S, obese mice with saline; O+L, obese with Liraglutide treatment; DO+S, diabesity mice with sline; DO+L, diabesity mice with Liraglutide treatment.

We examined BW and FBG in mice before and after administration with Liraglutide. Before Liraglutide treatment, the mice in the control group had lower BW compared with other mice. After Liraglutide treatment, the mice in the groups of O+L and DO+L had decreased BW compared with the groups of mice without treatment, that is, O+S and DO+S, respectively (Figure 1a). Furthermore, the mice of DO group had higher FBG levels compared with non-diabetic lean and obese mice. With Liraglutide treatment, they had decreased FBG levels compared with the mice of DO+S group (Figure 1b).

We also measured fasting serum insulin levels and found that the diabesity (DO) mice had increased fasting serum insulin levels after Liraglutide treatment. But the obese mice with Liraglutide treatment had decreased fasting serum insulin levels compared with obese mice fed by HFD and treated with saline (Figure 1c). We further analyzed HOMA-IR in mice. Data indicated that the mice in the groups of O+L and DO+L after Liraglutide treatment had decreased HOMA-IR compared with the mice of O+S and DO+S groups (Figure 1d).

### Hepatic AC3 gene expression at mRNA and protein levels in mice before and after Liraglutide treatment

**Figure 2:**
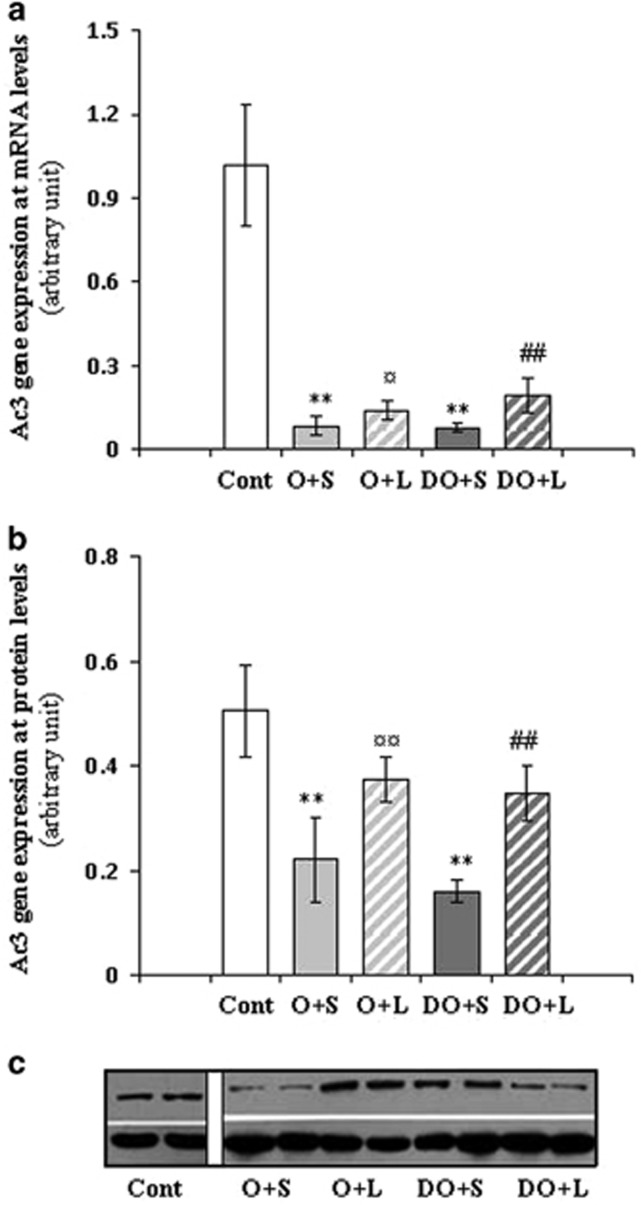
AC3 gene expression at mRNA and protein levels in the liver before and after Liraglutide treatment. AC3 gene expression at mRNA and protein levels in liver tissues of mice before and after Liraglutide treatment was shown in **a** and **b**, respectively. The image from western blotting experiments is represented in **c**. **P<0.001 versus the control group before Liraglutide treatment; ^¤^*P*<0.05 and ^¤¤^*P*<0.001 tests between O+S and O+L, while ^##^*P*<0.001 between DO+S and DO+L. Abbreviations: Cont, the control group of non-diabetic and lean mice; O+S, obese mice with saline; O+L, obese with Liraglutide treatment; DO+S, diabesity mice with sline; DO+L, diabesity mice with Liraglutide treatment.

We detected AC3 mRNA expression at both mRNA and protein levels in liver tissues and the results were summarized in Figure 2a–c. We found that AC3 gene expression at both mRNA and protein levels in obese, db/db and diabesity mice was significantly lower compared with the control mice, which were non-diabetic and lean. After Liraglutide treatment for 12 weeks, AC3 gene expression at both mRNA and protein levels in obese (O+L) and diabesity (DO+L) mice were increased compared with the mice of O+S and DO+S groups. Furthermore, AC3 mRNA expression levels were negatively correlated with BW (*r*=−0.882, *P<*0.05) and HOMA-IR (*r*=−0.682, *P<*0.05). We also examined AC3 protein expression in the liver of mice by western blotting. The alteration of AC3 expression at protein level was similar to AC3 mRNA expression in the liver. AC3 protein expression was negatively correlated with the BW (*r*=−0.657, *P<*0.05) and HOMA-IR (*r*=−0.756, *P<*0.05), suggesting a possible link between AC3 expression and insulin resistance.

### Histological and morphometric analyses of liver tissues in mice before and after Liraglutide treatment

We also examined the liver tissue samples with histological protocols. H&E staining revealed that in the liver of the control group of non-diabetic and lean mice, there was fewer lipid droplets and scattered inflammatory foci compared with those in O+S and OD+S groups (Supplementary Figure 1a). The numbers of lipid droplets and scattered inflammatory foci in the liver of the mice in O+L and OD+L groups were fewer compared with the mice in O+S and OD+S groups. Oil Red O staining analyses confirmed the improvement of hepatic histology after Liraglutide treatment (Supplementary Figure 1b). The area ratio of hepatic fat deposition to liver tissues showed the same results. Furthermore, the area ratio of hepatic fat deposition to liver tissue was positively correlated with BW (*r*=0.898, *P<*0.001) and HOMA-IR (*r*=0.836, *P<*0.001) and was negatively correlated with AC3 mRNA expression (*r*=−0.818, *P<*0.001). Liver steatosis, inflammation, ballooning and Nonalcoholic Steatohepatitis scores were all significantly higher in the mice of O+S and OD+S groups than in those of O+L and OD+L groups (Table 2). However, there was no significant change of liver fibrosis in mice among the groups (data not shown).

## Discussion

We comparatively analyzed hepatic AC3 gene expression at both mRNA and protein levels in obese and diabesity mice with and without Liraglutide treatment. With Liraglutide treatment, AC3 gene expression at both mRNA and protein levels was found to be increased, whereas BW, FBG levels and HOMA-IR index in the treated mice were decreased. Furthermore, the upregulation of AC3 gene expression was negatively correlated with the reduction of BW, FBG levels and HOMA-IR index.

Abdel-Halim *et al.* previously demonstrated that AC3 mRNA expression in pancreatic islets of GK rat was overexpressed compared with Wistar rat.^11^ GK rat is the spontaneously diabetic animal model and useful to study the defective β-cell function with resulting impairment of glucose-stimulated insulin release.^12^ In men, there is a co-dependent relationship between T2D and obesity,^27^ whereas the GK rat is diabetic but not obese. Therefore, the observation concerning the AC3 gene overexpression in pancreatic islets of GK rat implicated that this gene might have the susceptibility to T2D or protective effects in obesity. Later on, Nordman *et al.* conducted the first genetic association study in Swedish population and demonstrated that the AC3 genetic polymorphisms are not associated with T2D but confer a protective effect in obesity.^14^ The genetic association study was replicated in Chinese and European populations and the data are consistent.^15, 16^ In further support, Wang *et al.* have demonstrated that AC3 knockout mice develop obesity when aging.^17^ In the present study, we developed obese and diabesity mice by inducement with high-fat diet from C57BL/6 J and db/db mice, respectively. We also found that the hepatic AC3 gene expression at mRNA and protein levels in obese and diabesity mice was extremely low compared with non-diabetic and lean mice. Therefore, the present study provided further evidence that AC3 is an anti-obesity gene and has an important role in the regulation of BW.^18^

We had a hypothesis that AC3 gene expression might be activated by Liraglutide and therefore comparatively analyzed hepatic AC3 gene expression at both mRNA and protein levels in obese and diabesity mice with and without Liraglutide treatment. We observed the histological improvement in the liver of obese and diabesity mice after Liraglutide treatment. We also found that AC3 gene expression in treated obese and diabesity mice was upregulated by double fold compared with the untreated mice but far away from the recovery in non-diabetic and lean control mice. First, results from the present study supported our hypothesis that AC3 gene expression was upregulated by administration of Liraglutide. Second, the reduction of BW and blood glucose levels after Liraglutide treatment indicated that this drug has significantly independent glucose and weight reducing effect.^7^ The AC3 activation was only partially involved in the effect. In addition, we realized that data from real-time reverse transcription-PCR and western blotting experiments in the present study showed that the ranges of upregulation of AC3 gene expression at mRNA and protein levels in obese and diabesity mice after Liraglutide treatment were different. This may be explained by the cause of methodologies. Because there are nine closely related isoforms of ACs (AC1–9) in mammals, AC3 shares high homology with others.^8, 9^ In western blotting, the detection of AC3 protein with polycolonal antibody is most likely over-lapped with other isoforms. Recently, proximity ligation assay has been developed for protein analyses. This technology extends the capabilities of traditional immunoassays to include direct detection of proteins with high specificity and sensitivity.^28^ To avoid the crossover problem, development of single proximity ligation assay to analyze the AC3 protein has been taken into our consideration.

Taking together, the present study provides evidence supporting our hypothesis that AC3 gene is upregulated by administration of Liraglutide. The AC3 activation, however, contributes partially in reduction of BW and improvement of insulin resistance with Liraglutide treatment. Further investigation of the molecular mechanism has been taken into our consideration.

## Figures and Tables

**Table 1:** Classification of fibrosis in liver by nonalcoholic steatohepatitis clinical research network

*Fibrosis type*	*Score*
None	0
Perisinusoidal zone	3
Mild	1A
Moderate	1B
Portal/periportal	1C
Perisinusoidal and portal/periportal	2
Bridging	3
Cirrhosis	4

**Table 2:** Scores of fibrosis in the liver before and after the treatment with Liraglutide

	*Cont*	*O+S*	*O+L*	*OD+S*	*OD+L*
Steatosis score	0.00 ±0.00	2.75 ±0.46**	0.88 ±0.35^##^	2.75 ±0.46**	1.88 ±0.83^¤^
Inflammation score	0.75 ±0.46	2.00 ±0.76**	1.00 ±0.76^##^	2.00 ±0.76**	1.25 ±0.46^¤^
Ballooning score	0.00 ±0.00	1.13 ±0.35**	0.25 ±0.46^##^	1.13 ±0.59**	0.38 ±0.52^¤^
NASH score	0.25 ±0.46	5.88 ±0.86**	2.13 ±0.93^##^	5.88 ±0.64**	3.50 ±0.93^¤¤^

Abbreviations: Cont, control group; NASH, nonalcoholic steatohepatitis; O+S, obese alone+saline; O+L, obese with Liraglutide treatment; OD+S, diabetobese+saline; OD+L, diabetobses with Liraglutide treatment. Data are the means±s.e. Comparative analyses were performed as: and **P<0.01 versus the negative control group; ^##^*P<*0.01 versus the O+S group; ^¤^*P<*0.05 and ^¤¤^*P<*0.01 versus the OD+S group.

## Supplementary Material

Supplementary Figure1
