# Supplementary figures and images for: Hepatic adenylate cyclase 3 is upregulated by Liraglutide and subsequently plays a protective role in insulin resistance and obesity
Source: Nutr Diabetes. 2016 Jan 25;6(1):e191–. doi: 10.1038/nutd.2015.37 (PMC4742720; doi:10.1038/nutd.2015.37)

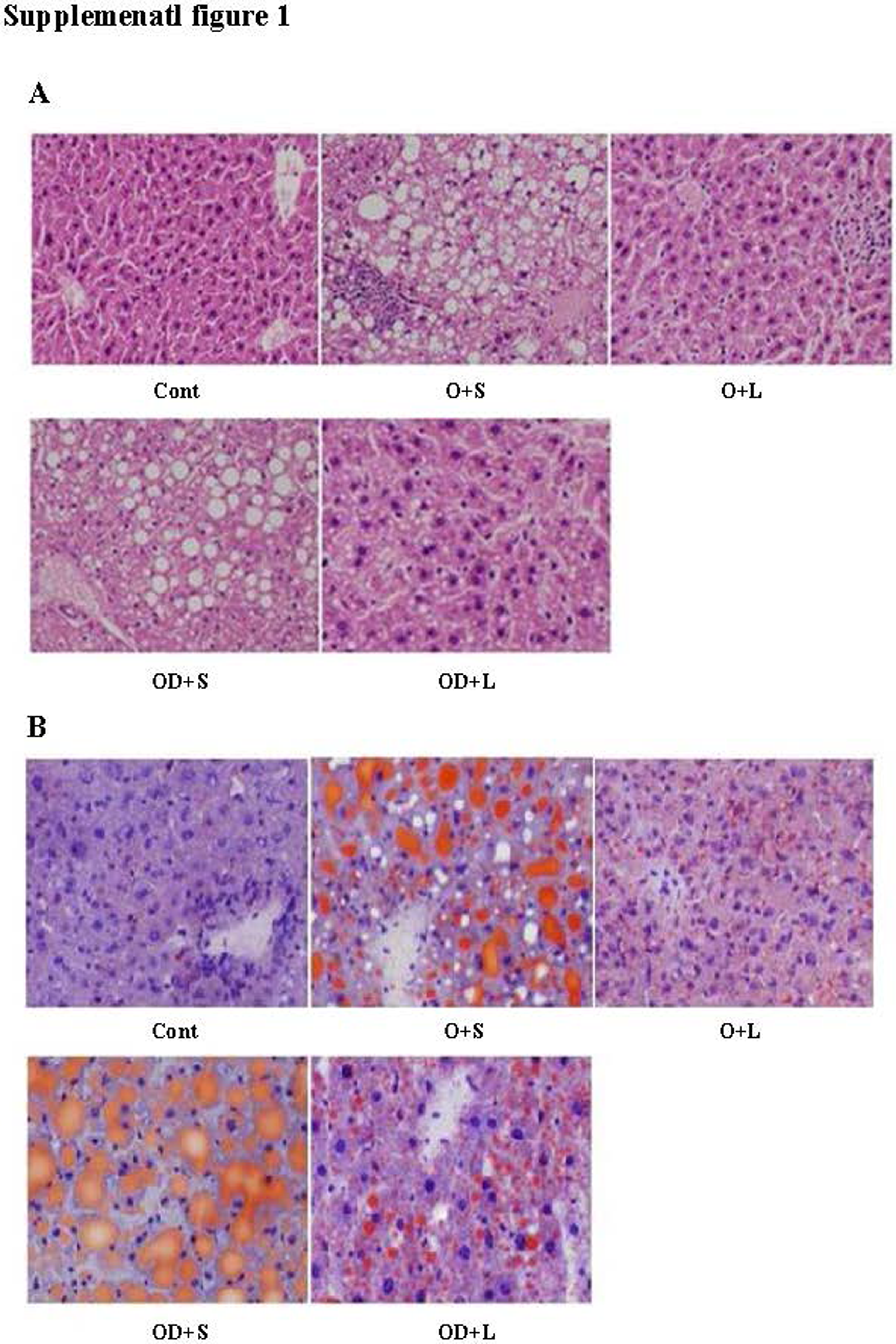

Supplement: Supplementary Figure1 [file nutd201537x1.tif]
